# Endothelial Dysfunction in Patients with Advanced Heart Failure Treated with Levosimendan Periodic Infusion Compared with Optimal Medical Therapy: A Pilot Study

**DOI:** 10.3390/life12091322

**Published:** 2022-08-26

**Authors:** Alessandro Maloberti, Jinwei Sun, Jessica Zannoni, Lucia Occhi, Ilaria Bassi, Saverio Fabbri, Valentina Colombo, Elena Gualini, Michela Algeri, Marisa Varrenti, Gabriella Masciocco, Enrico Perna, Fabrizio Oliva, Manlio Cipriani, Maria Frigerio, Cristina Giannattasio

**Affiliations:** 1 Cardiology 4, Cardio Center, ASST GOM Niguarda, Piazza Ospedale Maggiore 3, 20159 Milan, Italy; 2 School of Medicine and Surgery, University of Milano-Bicocca, 20126 Milan, Italy; 3 Cardiology 2, Cardio Center, ASST GOM Niguarda, 20126 Milan, Italy; 4 Cardiology 1, Cardio Center, ASST GOM Niguarda, 20126 Milan, Italy

**Keywords:** endothelial dysfunction, flow-mediated vasodilation, levosimendan, heart failure

## Abstract

Endothelial dysfunction (ED) is frequently found in patients with heart failure (HF). Among several pharmacological agents reported to improve endothelial function, levosimendan seems to be a promising one, even though, to date, only two previously published studies have evaluated its effects on ED in these patients. The aim of our pilot study was to further investigate the role of periodic levosimendan infusion on endothelial function in patients affected by advanced HF. In this cross-sectional study, three different groups were enrolled: 20 patients with advanced HF treated with periodic levosimendan (LEVO), 20 patients with HF on optimal medical therapy (OMT), and 20 healthy subjects (control group). ED was evaluated through flow-mediated dilation (FMD) at the level of the brachial artery. The three groups presented similar ages with significant differences in gender distribution, systolic blood pressure, and chronic kidney disease (eGFR < 30 mL/min). In HF patients, ischaemic aetiology was more prevalent in the LEVO group than in the OMT group (60 vs. 40%, *p* < 0.001). The New York Heart Association (NYHA) functional class was worse in the LEVO group, as well as in NT-proBNP (5636.7 ± 6164.6 ng/dL and 1243.7 ± 1487.2 ng/dL, in the LEVO and OMT groups, respectively, *p* = 0.005). The FMD was significantly higher in the healthy control group compared to that of the OMT group (15.7 ± 6.4 vs. 9.1 ± 6.0%, *p* = 0.007) while it showed an intermediate value in LEVO patients (12.4 ± 7.1%) (ANOVA *p* = 0.010). In conclusion, levosimendan therapy seems to ameliorate endothelial dysfunction related to heart failure. Longitudinal studies in patients on periodic therapy are needed in order to confirm the long-term effects of levosimendan on ED.

## 1. Introduction

In heart failure (HF) patients both structural and functional heart and vessel alterations account for its genesis and progression [1]. Among the various pathophysiological mechanisms, endothelial dysfunction (ED) seems to be significantly involved in both exacerbating the already existing vasoconstriction and increasing myocardial damage [2,3,4]. Among the several methods available for assessing endothelial function, flow-mediated vasodilation (FMD) at the level of the brachial artery evaluated via ultrasound methods is a commonly used and reliable noninvasive method. It is an endothelium-dependent function, based on the release of nitric oxide (NO), in response to reactive hyperaemia via increased shear stress, which results in vasodilation that can be quantified as an index of vasomotor function [5,6].

Several pharmacological agents were reported to improve endothelial function [7,8,9,10,11,12,13,14] and, among them, levosimendan (a calcium-sensitizing inotropic agent used for the management of HF) [15,16,17] presents vasodilatory properties through the opening of adenosine triphosphate (ATP)-sensitive potassium channels and the induction of endothelial NO production [18,19]. To date, only two previously published studies have evaluated its effects on ED in HF patients with positive results [20,21].

Our pilot study aimed at further investigating the role of periodic levosimendan therapy on endothelial function in patients affected by advanced HF. The data of these subjects were compared with those of a group of HF patients on optimal medical therapy (OMT) and a control group of healthy subjects.

## 2. Materials and Methods

### 2.1. Study Population

In this cross-sectional study, three different groups, with a total number of 60 subjects, were enrolled: 40 patients with advanced HF, 20 of whom were treated with periodic levosimendan infusion (LEVO) and 20 were on OMT, and 20 healthy subjects served as a control group. Patients were enrolled between January 2018 and February 2020.

Sample size calculations indicated the need for 20 patients per group in order to find a significant difference of 5.5% in FMD (with a standard deviation of 6%) with a power of 0.8 and a type 1 error of 0.05.

LEVO subjects were those who presented advanced symptomatic HF but who did not fulfil either the criteria for heart transplantation or the left ventricular assist device implantation. They were treated with levosimendan infused in 24 h at 0.05–0.1 µg/kg/min and repeated every four weeks. Patients were enrolled at least after their third levosimendan infusion in order to obtain a stable effect (steady state) on endothelial function.

The OMT group was composed of HF subjects who were on maximized medical therapy according to the ESC 2016 guidelines for the diagnosis and treatment of acute and chronic heart failure [1].

HF patients of both groups needed to be in a stable condition with no changes either in therapies or in functional status for at least 3 months. The functional status of the patients was assessed through the New York Heart Association (NYHA) class. Furthermore, an admission to hospital for HF in the previous 3 months was another exclusion criterion. All subjects were recruited among the patients under follow-up at the outpatient clinic of the Heart Failure Cardiological Department of our hospital.

Finally, the control group consisted of healthy volunteers with no previous history of cardiovascular (CV) diseases or therapies. They were recruited among the patients followed by the hypertensive unit of our hospital. All the subjects were without CV therapies at the time of the ambulatory visit and study enrolment. In some of them, a diagnosis of hypertension (6 subjects), dyslipidaemia (6 subjects) and Diabetes Mellitus (1 subject) was completed at the time of the visit. However, relative therapies had been started after the FMD evaluation.

### 2.2. Protocol

**Figure 1:**
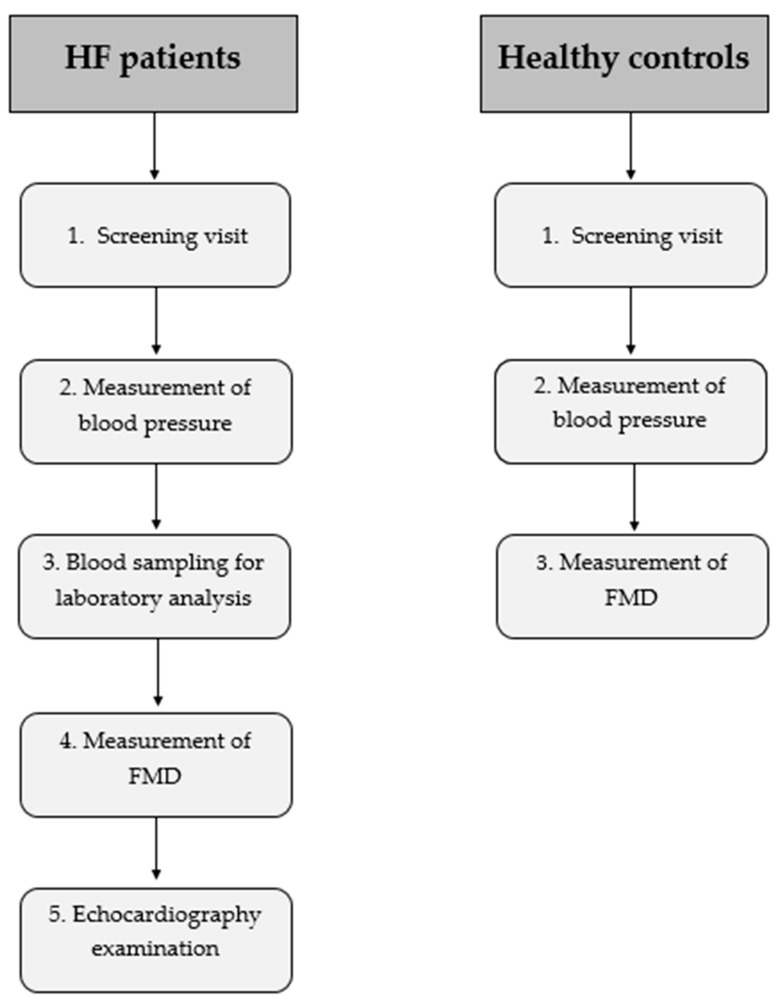
Protocol timeline flowchart. FMD = Flow-mediated dilation.

During the initial visit, a comprehensive medical history was collected and a complete physical examination was performed for all subjects. A timeline of the protocol is summarized in Figure 1.

Regarding medical history, particular attention was paid to collecting data regarding CV risk factors and, in HF patients, data related to aetiology, functional status (NYHA class), and therapies. Data regarding right heart catheterization after the third levosimendan infusion were collected in the LEVO group and data about the stable medical therapies were also collected for the OMT group.

After that, height and weight were obtained to calculate body mass index (BMI) and, with the patient in the supine position for at least 5 min and with the arm placed at heart level, blood pressure (BP) measurements were taken by a trained physician with an oscillometric automatic device (OMRON 705-IT, OMRON Healthcare Europe, Hoofddorp, Netherlands). BP was measured twice and the mean of the two measurements was recorded.

In HF patients, a blood sample was taken on the day of the visit to measure fasting serum glucose, serum total cholesterol, high-density lipoprotein (HDL) cholesterol, low-density lipoprotein (LDL) cholesterol, serum triglycerides, creatinine, and N-terminal prohormone of brain natriuretic peptide (NT-proBNP) levels. Glomerular filtration rate was estimated (eGFR) by the modification of diet in renal disease (MDRD) equation and chronic kidney disease (CKD) was defined as a value < 30 mL/min/1.73 m^2^.

After the blood sample, with the patient maintaining the supine position, FMD of the brachial artery was measured, followed by a complete echocardiographic examination (not performed in healthy controls). In the LEVO group, these evaluations were completed before the beginning of the new treatment, i.e., 4 weeks after the last levosimendan infusion.

### 2.3. Flow-Mediated Vasodilation

FMD ultrasound measurements of the brachial artery were performed according to relative guidelines [5,6]. Using a high-resolution ultrasound (ESA-OTE MyLabα, Genova, Italy) with a 7.5 MHz linear array transducer, the measurements of the right brachial artery diameters were taken after supine rest for at least 10 min and after the complete deflation of the cuff performing the 5 min supra-systolic compression (50 mmHg above systolic pressure) of the right upper arm. A stereotactical arm was used for optimal transducer positioning on the brachial artery proximal to the bifurcation of the radial and ulnar arteries. The longitudinal image of the artery was recorded at baseline and immediately after cuff deflation. The baseline and maximum FMD diameters were measured from one media-adventitia interface to the other of the artery at the end-diastole of the cardiac cycle with a real-time computerized edge detection system (Esaote, Genova, Italy) to obtain more precision and reproducibility. FMD of the brachial artery was estimated as the percent change in diameter over the baseline value.

The evaluation was completed by the same researcher for all the patients (AM). In our laboratory, the intrasession within- and between-operator variability of flow-mediated vasodilation were characterized by a coefficient of variation of the mean value amounting to 8.5 and 9.4%, respectively.

### 2.4. Cardiac Ultrasonography

Two-dimensional (2D) echocardiograms were performed by an experienced cardiologist using a dedicated ultrasound machine (with an ultrasound transducer of 2.5 MHz) for each HF patient. Two-dimensional high frame rate grey-scale loops of four-chamber, two-chamber, and three-chamber views with average frame rates of 50 frames per second (fps) were used in order to measure left ventricular end-diastolic diameter (LVEDD), interventricular septum and posterior wall thickness; left ventricular ejection fraction (LVEF) was evaluated using the Simpson method.

Mitral diastolic inflow was interrogated using pulsed-wave Doppler from the apical four-chamber view with the sample volume placed at the level of the mitral leaflet tips. Mitral early diastolic peak (E wave) and late peak (A wave) velocities, E and A ratio, and deceleration time of mitral early velocity were measured. Tissue Doppler (TD) was obtained at the apical four-chamber view with the sample volume placed at the lateral mitral annulus. Early diastolic mitral annulus peak velocity (e’) was registered, and the ratio of transmitral diastolic peak velocity to the mitral annular diastolic peak velocity (E/e’) was calculated.

Finally, the function of the right ventricle was evaluated through the tricuspid annular plane systolic excursion (TAPSE) index as well as s’ value with TD at the tricuspid level. 

### 2.5. Statistical Analysis

The results were expressed as mean ± standard deviation and median (interquartile range) for continuous normally distributed and non-normally distributed (triglycerides and NT-pro-BNP) variables, respectively. The percentage was used to describe categorical variables. Between-group differences were assessed by Student *t*-tests, Mann–Whitney tests, and χ^2^ tests (or a Fisher exact test when needed) for normally distributed, non-normally distributed and categorical variables, respectively. When more than two groups were analysed, the ANOVA test was employed. Bonferroni correction was used in order to evaluate differences between groups. 

Assessment of correlations was completed using Pearson product-moment correlation coefficient or Spearman’s rank correlation coefficient as appropriate. Multivariable linear regression models were performed with FMD as the dependent variable. Independent predictors were selected including the variables characterized by statistically significant Pearson correlation coefficients.

The statistical analysis was carried out using IBM SPSS Stat Editor software and a *p*-value < 0.05 was considered statistically significant.

## 3. Results

Table 1 reports the clinical, laboratory, and echocardiographic characteristics and FMD of the three evaluated groups.

The three groups presented similar age, BMI, and heart rate (HR) while the male sex was prevalent in OMT and LEVO groups when compared with healthy controls (80, 85, and 50% respectively, *p* = 0.029). Regarding CV risk factors, systolic BP (SBP) was significantly lower in OMT and LEVO groups when compared to healthy controls (97.0 ± 12.2, 102.0 ± 12.4, and 118.0 ± 18.6 mmHg, respectively, *p* < 0.001) while there were no differences in the prevalence of hypertension, dyslipidaemia, and diabetes mellitus, apart from the smoking status (*p* = 0.028). CKD was more prevalent in the LEVO group (50, vs. 20% in the OMT group and 0% in healthy controls, *p* = 0.001).

In HF patients we found a significant difference regarding etiology with the ischaemic cardiomyopathy more prevalent in the LEVO group than in the OMT group (60 vs. 40%, *p* < 0.001). NYHA functional class was worse in patients with levosimendan, in fact, 95 vs. 45% of patients were in class three or four while 5 vs. 45% were in class two (*p* = 0.001). In addition, NT-proBNP was significantly higher in LEVO patients (755.5 vs. 4217 ng/dL, *p* = 0.005). Both groups presented with a similar and very long heart disease history (132 months, 11 years), patients on levosimendan started the therapy on average 22 ± 26 months before our evaluation.

Regarding echocardiographic variables, significant differences were found for E and A, E/e’, indexed left atrial volume, and systolic pulmonary arterial pressure (sPAP), which were all significantly higher in the LEVO group than in the OMT group. LVEF was lower in LEVO patients, although the difference did not reach statistical significance (23.5 ± 6.1 vs. 27.8 ± 7.8%, *p* = 0.057).

Finally, patients on levosimendan showed at right heart catheterization a higher mean pulmonary artery pressure and pulmonary vascular resistance index with lower wedge pressure when compared to patients on OMT, while the cardiac index was superimposable.

Table 2 shows the CV therapies of the two HF groups. OMT patients took the angiotensin receptor-neprilysin inhibitor more frequently than those of the LEVO group (90 vs. 15%, *p* < 0.001) with no significant differences regarding the mean dose. LEVO patients used angiotensin-converting enzyme inhibitors (ACE-I) more frequently (50 vs. 5%, *p* = 0.003). Despite similar prevalence in loop diuretic use, patients on levosimendan took higher doses of furosemide (148.6 ± 91.7 vs. 62.5 ± 61.9, *p* = 0.002). No other significant differences were found regarding drug class and dosage. 

**Figure 2:**
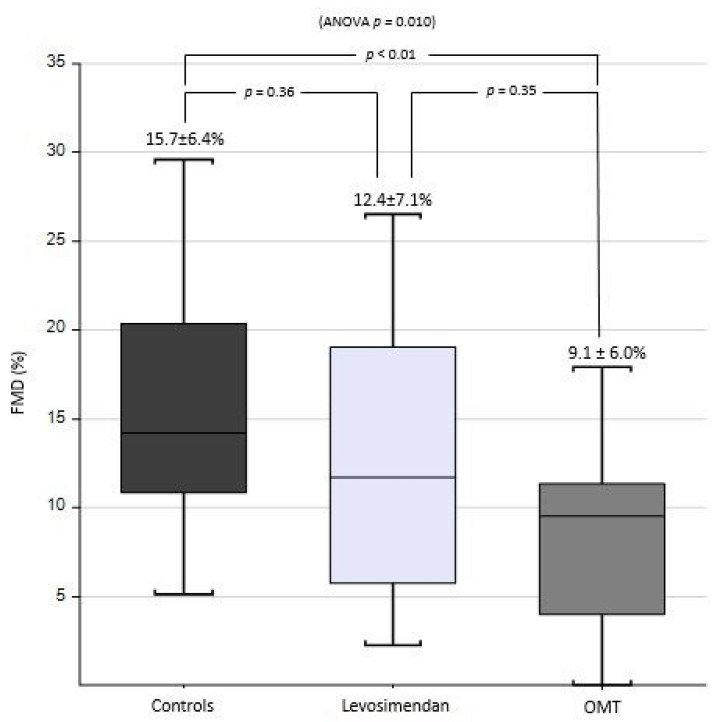
Flow-mediated dilation (FMD) in percentage in optimal medical therapy (OMT), levosimendan and healthy control groups.

Finally, as shown in Figure 2, FMD was significantly higher in the healthy control group compared to that of the OMT group (15.7 ± 6.4 vs. 9.1 ± 6.0%, Bonferroni *p* = 0.007) while an intermediate value was found in LEVO patients (12.4 ± 7.1%). The overall ANOVA *p*-value was 0.010. A Bonferroni analysis indicated that no further significant differences were found (neither between LEVO and OMT groups nor between LEVO and healthy subjects). 

At univariate analysis FMD correlates with sex (r = 0.287; *p* = 0.026), CKD (r = −0.325; *p* = 0.011), and being of the OMT (r = −0.334; *p* = 0.009) and control groups (r = 0.336; *p* = 0.009).

A linear regression model with FMD as the dependent variable and age, sex, CKD, and patients’ group (OMT, LEVO, and healthy controls) was performed (Table 3).

Using the LEVO group as a reference, we found that CKD and being a part of the OMT group are significant determinants of FMD. These associations present negative β values and, therefore, a significant association with lower FMD values is present. On the contrary, these results confirm that LEVO patients present similar values to healthy controls also on multivariate analysis.

## 4. Discussion

Our study demonstrated that patients on levosimendan periodic therapy at a steady state had an intermediate value of FMD at the level of the brachial artery (12.4 ± 7.1%), between that of the OMT group (9.1 ± 6.0%) and that of the healthy control group (15.7 ± 6.4%). Results of a significant difference between the LEVO group and the OMT group with values similar to the healthy subjects have been found also through multivariate analysis. Despite the limits determined by the cross-sectional nature, we can infer from our results that levosimendan could be able to partially improve ED in HF patients. This result has been found despite the LEVO group having a more deteriorated heart function (high ischaemic aetiology prevalence, NYHA class, NT-proBNP, intraventricular filling pressure and sPAP, low LVEF, higher mean pulmonary artery pressure and pulmonary vascular resistance index, higher furosemide dose). Although in this condition we would expect to find a heavily impaired endothelial function, this was not the case, confirming the important effect of levosimendan on endothelial function.

Our results agree with those of two previously published studies on the topic. Parissis et al. have demonstrated that levosimendan improved endothelial function and reduced the detrimental activation of adhesion molecules in advanced chronic HF patients in a randomized study on 26 patients. After 48 h from levosimendan infusion, a significant improvement of FMD (from 4.8 ± 3.0% to 6.4 ± 4.4%, *p <* 0.05) with concomitant reduction of plasma concentrations of adhesion molecule was observed [20]. In another prospective observational trial that involved adult cardiogenic shock patients (10 total subjects) supported with venoarterial extracorporeal life support the use of levosimendan was shown to improve endothelial function and hemodynamics and facilitate weaning from the extracorporeal support. In particular FMD increased from 3.2 ± 4.2% to 17.8 ± 10.4% (*p* < 0.001) when reassessed immediately after the end of the 24 h infusion [21].

Therefore, these two studies demonstrated that a levosimendan infusion was able to determine an improvement in FMD immediately after its end [21] and that these positive effects could last at least 48 h [20]. In both studies, patients were unaware of the levosimendan treatment and were in an acute setting (refractory cardiogenic shock [21] and acute HF [20]). Furthermore, FMD was evaluated only after the first levosimendan dose.

Our study is able to add new important pieces of information regarding the long-term effects of levosimendan infusions in the context of chronic advanced HF subjects. In fact, because our FMD evaluation was completed 4 weeks after the last levosimendan infusion (before the new planned infusion) we can deduce that levosimendan could determine positive long-term effects when infused periodically. Many potential mechanisms could explain our results. Levosimendan performs as an inodilator through a tripartite mechanism which involves acting as a calcium sensitizer in cardiomyocytes by increasing the sensitivity of troponin C fibres to ionic calcium and as a vasodilator and cytoprotective agent through the opening of ATP-dependent potassium channels on vascular smooth muscle cells and in mitochondria [19]. Thus, the hemodynamic effects of levosimendan have been attributed to the combined effect of increased myocardial contractility and peripheral vasodilation, the latter leading to decreases in both preload and afterload. In addition, it also exerts a variety of cardioprotective actions in chronic HF, such as anti-inflammatory, antioxidant, and antiapoptotic actions, by activating ATP-dependent potassium channels in both the plasma membrane and the mitochondrial matrix of cardiac myocytes [22,23]. Therefore, the beneficial effects of levosimendan on FMD and endothelial function could be attributed to the increase in blood flow, induced not only by the enhancement of cardiac output in the short term but also by the cardioprotective activity in the long term, which in turn leads to elevation of shear stress.

Some other points of our study deserve to be mentioned, i.e., the confirmation of the presence of ED in HF patients as well as the reduced efficacy of OMT on its reversion.

As mentioned previously, ED has been found in HF subjects [2,3,4,24]. Furthermore, it presents an important prognostic role in future end-point [25,26,27]. Our paper confirmed the presence of ED in HF patients as demonstrated by their lower FMD values in comparison with the healthy control group.

Various drug treatments demonstrated to ameliorate ED in HF subjects, such as ACE-I [28], statins [9,10,11], ivabradine [12], and allopurinol [10]. Additionally, in this case principally acute effects on FMD were evaluated (from a few days to some weeks with only one study with a 6-month follow-up time [9]). 

Focusing on ACE-I, the BANFF study [29] compared the effect on endothelium dysfunction in 80 patients treated with quinapril, enalapril, losartan, and amlodipine. In this crossover study, quinapril was associated with a significant improvement in FMD. 

The PERFECT [30] trial was a 3-year double-blind randomized placebo-controlled study that analysed the effect of 8 mg of perindopril once daily on FMD in patients with stable ischaemic heart disease without HF. At 36 months, perindopril resulted in a modest, albeit not statistically significant, improvement in FMD.

A meta-analysis of randomized controlled trials performed by Shahin et al. [7] showed that ACEIs improved brachial FMD in patients with endothelial dysfunction caused by various conditions and that they were superior to CCBs and β-blockers. No significant difference between the effect of ACEIs and ARBs was found in this study.

Okutucu et al. [31] investigated angiotensin receptor neprilysin inhibitors (ARNI) and enrolled a total of 25 patients with HF with reduced LVEF. Endothelial function was measured at baseline and one month after ARNI treatment by brachial artery FMD. The study population was found to significantly improve after ARNI treatment (2.69 ± 1.93% vs. 6.83 ± 2.87%, *p*< 0.001). Improvements of FMD were correlated with clinical improvement and NT-proBNP.

Finally, Nathaniel et al. [32] analysed 10 stable HF with reduced LVEF patients starting ARNI comparing them with another 10 stable patients with the same condition but that remained on conventional treatment. FMD increased after 12 weeks of ARNI (2.2 ± 1.9 vs. 5.5 ± 2.1%; *p* < 0.001) but not in the controls (4.8 ± 3.8 vs. 5.4 ± 3.4%; *p* = 0.34). 

Data regarding the effects of OMT on endothelial function, particularly its long-term effects, are lacking. Based on our data, OMT seems not to be effective in reducing ED or at least seems to be less effective than levosimendan. As already mentioned, LEVO patients had a more deteriorated heart function when compared to the OMT group with higher ischaemic aetiology prevalence, NYHA class, NT-proBNP, intraventricular filling pressure and sPAP, and lower LVEF. This further confirms the important effect of levosimendan on endothelial function and the minimal effect of OMT.

Left ventricular assistance device (LVAD) is a possible nonpharmacological therapy for subjects with advanced HF. These devices can draw blood from the left ventricle and pump it directly into the aorta, supplanting the depressed function of the left heart [33]. The latest generation of devices consists of rotating continuous flow pumps, of limited size, able to generate a range of up to 10–12 L/min of continuous flow [34]. One potential adverse effect of long-term continuous-flow LVAD support is arterial endothelial cell dysfunction that could result in impaired vascular reactivity. After the implementation of LVAD circulatory support, the pulsatile nature of the arterial flow pattern decreases dramatically. In addition to the longitudinal stretching forces, the so-called “shear stress”, the cyclical deformation produced by the pulsatility of the flow represents an independent modulator of the endothelial function which is able to exert an impact on nitric oxide synthase, cell Ph, and blood cell physical alignment. Pulsatile shear stress and cyclic strain of an appropriate magnitude are required to maintain endothelial cell homeostasis [35]. While for the first generation of pulsatile flow LVADs the favourable effects on the endothelial function have been widely demonstrated, for the ones with continuous flow, heterogeneous data are emerging but contrasting.

A study that evaluated endothelial function by FMD in subjects with LVAD, comparing them with HF subjects and patients with previous heart transplantations demonstrated no significant effect of continuous flow LVAD on endothelial function [2]. Similar results were also found by Morgan et al. [36] with no significant differences in FMD among 20 patients with LVAD, 19 patients with HF, and 19 patients with previous heart transplantations. Symons et al. [37] showed no effect of durable-continuous-flow LVAD support on coronary artery endothelial function and even an improvement in subjects with nonischaemic dilated cardiomyopathy, using ex vivo isometric tension procedures among 16 patients with ischaemic cardiomyopathy, 22 patients with nonischaemic cardiomyopathy, and in seven controls. In contrast, Hasin et al. [38] evaluated the reactive hyperaemia index in eight subjects with HF before and after (5–14 days, 1–2 months, and 3–6 months) the LVAD implantation. The study showed a progressive decline in the hyperaemia index and therefore a worsening of endothelial function related to the LVAD.

Our study has some limitations. The main limitation is the cross-sectional nature of the study that prevented us from collecting longitudinal information on the FMD changes at different timepoints during an interinfusion period as well as its long-term effects after many infusions. Furthermore, only a few subjects were enrolled for every group. However, evaluation of ED through FMD is a complicated method in which risks of error are frequent and need to be completed accurately by the same operator in all the subjects.

Another important limitation is that vascular dysfunction can also be an endothelium-independent function via alterations in vascular structure and smooth muscle cell function rather than changes in endothelial cells. The major modus operandi of levosimendan is that this drug can open ATP-sensitive potassium channels in vascular smooth muscle cells, and the vascular dilatory effects of the drug lead to a decreased preload and afterload, putting less work on the heart. Therefore, there is a significant impact of levosimendan on vascular smooth muscle cell and endothelium-independent vasodilation can be useful to evaluate this point. In fact, responses to exogenous NO donors (e.g., glyceroltrinitrate) or vasodilators acting directly on vascular smooth muscle (e.g., adenosine) can be compared to differentiate endothelium-dependent from endothelium-independent responses [6]. In our study, we did not evaluate the endothelium-independent nitroglycerin-mediated vasodilation, because of the fragility of the enrolled patients and the possibility of clinical complications due to exogenous NO donors. 

Additionally, we did not collect data on left heart hemodynamics (which did not permit us to couple vascular and cardiac levosimendan effects) and we did not perform any biomarker analysis (myeloperoxidase and NO, for example). Among the biomarkers, myeloperoxidase was found to be inhibited by levosimendan which is important in regulating endothelial function and vascular tone [39] and should be further tested in future studies. Furthermore, cardiopulmonary exercise test data were also lacking. Finally, although realistically normal, laboratory and echocardiographic data of the healthy control subjects were lacking.

Finally, as in any nonrandomized study, unmeasured bias could be a further potential source of error. In a pilot study, no further sensitivity analysis was possible [40,41] and this reinforced the need for further prospective trials in order to confirm our results.

## 5. Conclusions

In conclusion, chronic levosimendan periodic therapy is associated with better endothelial function when compared with OMT HF patients and with values that were similar to that of a control group. Our study is a pilot study and combining these results with the ones from the studies published so far, some unanswered questions still arise, i.e., does the FMD change in the days following the infusion until the next infusion or are its effects stable over time? Do these effects persist unchanged long term in levosimendan chronic use or is there an adaptation? Does the vascular effect move together with the heart inotropic one? Is OMT ineffective in ameliorating ED long term despite single drugs demonstrating an increase in FMD? Further prospective clinical trials are needed to evaluate the progressive benefits of levosimendan on ED in HF patients during the periodic infusions and to evaluate the role of long-term OMT on endothelial function.

## Figures and Tables

**Table 1:** Clinical, laboratory, echocardiographic and right heart catheterization data of the three groups of evaluated patients (OMT, levosimendan and healthy control).

	OMT	Levosimendan	Healthy Control	*p* Value (ANOVA)	*p* Value (OMT vs. LEVO)
Number	20	20	20	-	-
Clinical and anamnestic variables					
Age (years)	56.9 ± 8.3	59.1 ± 5.2	56.3 ± 11.2	0.561	0.975
Sex (male)	16 (80)	17 (85)	10 (50)	**0.029**	0.785
BMI (kg/m^2^)	26.1 ± 4.0	24.8 ± 3.2	26.4 ± 4.1	0.356	0.796
SBP (mmHg)	97.0 ± 12.2 *	102.0 ± 12.4 *	118.0 ± 18.6	**<0.001**	0.875
DBP (mmHg)	61.5 ± 6.3 *	67.9 ± 8.8	73.3 ± 11.6	**0.001**	0.095
HR (bpm)	65.5 ± 8.6	69.3 ± 8.2	71.7 ± 13.7	0.178	0.787
NYHA Class (%)	-	-	-	-	**0.001**
2	11 (55)	1 (5)	-	-	-
3	9 (45)	17 (85)	-	-	-
4	0 (0)	2 (10)	-	-	-
Etiology (%)	-	-	-	-	**<0.001**
Ischaemic	8 (40)	12 (60)	-	-	-
Nonischaemic	12 (60)	8 (40)	-	-	-
Disease duration (months)	132.6 ± 92.1	132.1 ± 147.3	-	-	0.24
Time from first levosimendan infusion (months)	-	22 ± 26	-	-	-
Smoke (%)	-	-	-	**0.028**	0.135
Nonsmoker	9 (45)	10 (50)	15 (75)	-	-
Current smoker	1 (5)	2 (10)	4 (20)	-	-
Former smoker	10 (50)	8 (40)	1 (5)	-	-
Hypertension (%)	2 (10)	3 (15)	6 (30)	0.235	0.334
Dyslipidaemia (%)	3 (15)	2 (10)	6 (30)	0.259	0.472
DM (%)	3 (15)	5 (25)	1 (5)	0.208	0.361
CKD (%)	4 (20)	10 (50)	0 (0)	**0.001**	0.067
Laboratory analysis					
Creatinine (mg/dL)	1.21 ± 0.36	1.48 ± 0.50	-	-	0.055
NT-proBNP (ng/L)	755.5 (540.7–1001)	4217 (2102.7–6329.7)	-	-	**0.005**
Glycemia (mg/dL)	91.9 ± 11.3	116.0 ± 24.8	-	-	**<0.001**
Total Cholesterol (mg/dL)	154.0 ±34.7	165.3± 51.8	-	-	0.422
LDL-C (mg/dL)	87.0 ± 29.0	96.6 ± 37.9	-	-	0.374
HDL-C (mg/dL)	42.7 ± 13.0	43.2 ± 17.5	-	-	0.919
Triglyceride (mg/dL)	132.5 (85.7–169.2)	115.5 (86–158.5)	-	-	0.349
Echocardiographic data					
LVEF (%)	27.8 ± 7.8	23.5 ± 6.1	-	-	0.057
E and A	1.5 ± 1.2	3.2 ± 1.8	-	-	**0.032**
MV DecT (ms)	171.0 ± 44.6	156.8 ± 31.5	-	-	0.521
E/e’	12.8 ± 7.2	19.2 ± 3.7	-	-	**0.034**
LA Volume Indexed by BSA (mL/m^2^)	53.0 ± 20.9	73.0 ± 15.8	-	-	**0.003**
TAPSE (mm)	16.9 ± 29.6	15.3 ± 3.2	-	-	0.152
Tricuspid lateral s’ (cm/s)	8.3 ± 1.4	7.3 ± 1.5	-	-	0.134
sPAP (mmHg)	32.7 ± 7.9	42.8 ± 12.9	-	-	**0.007**
Right Heart Catheterization data					
PAPm (mmHg)	22.9± 7.3	33.4 ± 11.2	-	-	**0.004**
WP (mmHg)	22.5 ± 7.09	15.1 ± 6.4	-	-	**0.005**
CI (L/min/m^2^)	2 ± 0.3	1.8 ± 0.4	-	-	0.142
PVRI (woods unit/m^2)^	3.9 ± 1.2	6.7 ± 5.2	-	-	**0.038**

* *p* < 0.05 versus control group. Data are reported as mean ± standard deviation and median (interquartile range) for continuous normally distributed and non-normally distributed (triglycerides and NT-pro-BNP) variables, respectively. BMI = body mass index; CKD = chronic kidney disease stage 4 and stage 5; DBP = diastolic blood pressure; DM = diabetes mellitus; FMD = flow-mediated vasodilation; HDL-C = high-density lipoprotein cholesterol; HR = heart rate; LA = left atrial; BSA = Body Surface Area; LVEF = left ventricle ejection fraction; LDL-C = low-density lipoprotein cholesterol; MV DecT = mitral valve deceleration time; NT-proBNP = N-terminal prohormone of brain natriuretic peptide; NYHA class = New York Heart Association class; SBP = systolic blood pressure; sPAP = systolic pulmonary artery pressure; s’ = pulsed doppler s wave; TAPSE = tricuspid annular plane systolic excursion; PAPm = pulmonary artery pressure mean, WP = wedge pressure; CI = cardiac index, PVRI = pulmonary vascular resistance index.

**Table 2:** Therapies of the HF groups (OMT and levosimendan).

	OMT	Levosimendan	*p*-Value
Number	20	20	
ASA (%)	10 (50)	8 (40)	0.751
Warfarin (%)	6 (30)	12 (60)	0.191
NAOC (%)	3 (15)	0 (0)	-
Beta-blocker (%)	20 (100)	16 (80)	0.106
Mean bisoprolol dose	6.6 ± 2.8	5.0 ± 1.5	0.645
ACE-I (%)	1 (5)	10 (50)	**0.003**
ARB (%)	1 (5)	5 (25)	0.182
ARNI (%)	18 (90)	3 (15)	**<0.001**
Mean sacubitril dose	51.4 ± 27.1	32.3 ± 14.4	0.264
MR antagonist (%)	17 (85)	17 (85)	1.000
Mean spironolactone dose	35.1 ± 20.7	42.9 ± 19.3	0.280
Loop diuretics (%)	18 (90)	19 (95)	1.000
Mean furosemide dose	62.5 ± 61.9	148.6 ± 91.7	**0.002**
Amiodarone (%)	5 (25)	12 (60)	0.054
Digoxin (%)	1 (5)	6 (30)	0.091
Ivabradine (%)	4 (20)	1 (5)	0.342
Statins (%)	12 (60)	10 (50)	0.751
Antidiabetic (%)	3 (15)	3 (15)	1.000

ACE-I = angiotensin-converting enzyme inhibitor; ARB = angiotensin receptor blocker; ARNI = angiotensin receptor-neprilysin inhibitor; ASA = acetylsalicylic acid; MR antagonist = mineralocorticoid receptor antagonist; NOAC = novel oral anticoagulant.

**Table 3:** A linear regression model with FMD as the dependent variable.

	β	95% Confidence Interval		*p*-Value
		Lower Bound	Upper Bound	
Age (years)	0.068	−0.141	0.251	0.575
Sex (female)	0.179	−1.176	6.646	0.167
CKD (present)	−0.302	−9.333	−0.494	**0.030**
LEVO group (reference)	-	-	-	-
OMT group	−0.324	−8.925	−0.537	**0.028**
Healthy controls group	0.002	−0.141	0.251	0.575

FMD = flow-mediated dilation; CKD = chronic kidney disease; OMT = optimal medical therapy.

## Data Availability

The authors declare that there is no conflict of interest. The data that support the findings of this study are available from the corresponding author upon reasonable request. We adhere to guidelines for rigorous reports of methods and results.
